# Melanoma screening: Informing public health policy with quantitative modelling

**DOI:** 10.1371/journal.pone.0182349

**Published:** 2017-09-25

**Authors:** Stephen Gilmore

**Affiliations:** 1 Skin and Cancer Foundation, Melbourne, Australia; 2 Dermatology Research Centre, Diamantina Institute, University of Queensland, Brisbane, Australia; Stony Brook University, UNITED STATES

## Abstract

Australia and New Zealand share the highest incidence rates of melanoma worldwide. Despite the substantial increase in public and physician awareness of melanoma in Australia over the last 30 years–as a result of the introduction of publicly funded mass media campaigns that began in the early 1980s –mortality has steadily increased during this period. This increased mortality has led investigators to question the relative merits of *primary* versus *secondary* prevention; that is, sensible sun exposure practices versus early detection. Increased melanoma vigilance on the part of the public and among physicians has resulted in large increases in public health expenditure, primarily from screening costs and increased rates of office surgery. Has this attempt at secondary prevention been effective? Unfortunately epidemiologic studies addressing the causal relationship between the level of secondary prevention and mortality are prohibitively difficult to implement–it is currently unknown whether increased melanoma surveillance reduces mortality, and if so, whether such an approach is cost-effective. Here I address the issue of secondary prevention of melanoma with respect to incidence and mortality (and cost per life saved) by developing a Markov model of melanoma epidemiology based on Australian incidence and mortality data. The advantages of developing a methodology that can determine constraint-based surveillance outcomes are twofold: first, it can address the issue of effectiveness; and second, it can quantify the trade-off between cost and utilisation of medical resources on one hand, and reduced morbidity and lives saved on the other. With respect to melanoma, implementing the model facilitates the quantitative determination of the relative effectiveness and trade-offs associated with different levels of secondary and tertiary prevention, both retrospectively and prospectively. For example, I show that the surveillance enhancement that began in 1982 has resulted in greater diagnostic incidence and reduced mortality, but the reduced mortality carried a significant cost per life saved. I implement the model out to 2028 and demonstrate that the enhanced secondary prevention that began in 1982 becomes increasingly cost-effective over the period 2013–2028. On the other hand, I show that reductions in mortality achieved by significantly enhancing secondary prevention beyond 2013 levels are comparable with those achieved by only modest improvements in late-stage disease survival. Given the ballooning costs of increased melanoma surveillance, I suggest the process of public health policy decision-making–particularly with respect to the public funding of melanoma screening and discretionary mole removal–would be better served by incorporating the results of quantitative modelling.

## Introduction

Melanoma is a major public health concern in Australia and, alongside New Zealand, has the highest incidence rates worldwide. Public awareness of the skin cancer risk associated with ultraviolet light exposure increased dramatically following the introduction of the ‘*Slip*, *Slop*, *Slap*’ campaign in Australia in 1982 [1, 2]. This campaign was also implemented in the United States. The messages of the campaign were twofold: first, to promote sensible sun exposure practices; and second, to encourage skin self-awareness with respect to skin cancer. The former corresponds to *primary* prevention of melanoma.

Formally, and after Dos Santos Silva [3], the following prevention strategies are defined: (i) Primary–*the prevention of the onset of melanoma*; (ii) Secondary–*the prevention of symptomatic melanoma*; and (iii) Tertiary–*the prevention of premature melanoma death*. Secondary prevention is thus synonymous with early detection and largely achieved via physician based melanoma screening. Screening can target either the general population or higher-risk subsets and involves full-body examinations performed regularly. Note that self-screening and encouraging melanoma awareness is also a secondary prevention strategy and will be promoted by public health campaigns primarily directed at primary prevention.

Melanoma awareness campaigns are likely to have contributed to the increased numbers of melanocytic lesions removed by medical practitioners over the last few decades. Indeed, evidence suggests that implementing skin cancer public awareness campaigns increases the excision rate of benign lesions [4]. While biopsy rates in the United States increased by 50% between 2002–2008, melanoma rates over the same period only increased by 4% [5]. The increase in the number of lesions excised over the last three decades is likely to be due to two factors working in concert: first, an increased presentation rate; and second, a lower threshold for excision.

Are too many pigmented lesions excised? Controversy has existed over the concept of *Number Needed to Treat* (NNT), a term loosely used to describe the number of benign naevi excised per melanoma excised [6]. For non-dermatologists, its value has been reported as 19.6 [7], 23 [8] and 22 [9]. While there may exist some debate regarding the pigmented lesion diagnostic abilities of dermatologists and general practitioners [10], it is possible the NNT for dermatologists may be lower–values of 6.3 [11] and 6.5 [12] have been reported. The controversy regarding the NNT relates to the meaning associated with its numerical value–is it better for it to be lower or higher? While a high NNT might indicate a practitioner is less likely to miss a melanoma, it could also mean that many lesions are removed unnecessarily, with the associated morbidity and increased costs. On the other hand, a low NNT might mean that melanomas are missed. Increased surveillance for melanoma coupled to high NNTs may result in large numbers of lesions removed without any significant reduction in mortality. However, there exists potential for physicians to optimise the value of their NNTs by either up-skilling and/or the utilisation of modern technology; for example, telemedicine or machine learning [13].

A corollary to the NNT is the *Number Needed to Screen* (NNS), a measure that reports on the number of people that need to be screened for a particular disease to save one life from that disease. It has been suggested that melanoma does not fare well in comparison with other life-threatening conditions. Estimates for melanoma extend to 25,000 screening assessments per life saved; in comparison, for example, the NNS for colon cancer may be around 800, and for preventing a cardiovascular death, the number of people needed to be screened for cholesterol levels may be as low as 480 [14].

Despite the increased public awareness of melanoma, and the large increase in the number of potential melanomas being excised, the mortality rate from melanoma has not declined in Australia over the last 30 years. Importantly, however, the incidence rate, while steadily increasing since 1982, has recently reached a plateau, and, for younger cohorts, is now in decline [15].

Intuitively one might expect that increased rates of surveillance can only lead to the desirable outcome of more lives saved since more early melanomas will be removed. Although physician-detected melanomas are thinner than those detected by patients [16], the potential difficulties in melanoma diagnosis are well documented [17]. Current evidence is insufficient to draw conclusions regarding the effects of melanoma surveillance on mortality [18, 19]. This lack of conclusive evidence suggests that the detection of early melanoma may not be critical. Indeed, it is possible that early melanomas will eventually declare themselves as changing lesions and thus can be excised before they have metastasized. There exist three main reasons why secondary prevention strategies are possibly limited in effectiveness. First, there will exist limits to the fraction of the population that will be screened. Second, even if screening is limited to high-risk individuals, and a large proportion of these individuals are screened, it is known that a significant fraction of melanoma deaths are due to melanomas that present in an advanced state; in contrast to the usual slowly developing lesions where early detection may not be critical, the more aggressive tumours demand early detection. However, because of their rapid evolution the latter may not be detected with regular screening. And finally, the progression of appropriately treated thin melanoma to advanced disease will occur in 5% of all patients [20].

Over-diagnosis confounds the relationship between incidence and mortality, and increases morbidity without any impact on mortality. For many types of human cancers it appears that over-diagnosis rates are problematic [21]. With respect to melanoma, it is likely that many excised lesions, diagnosed as melanoma, would not have otherwise eventually led to death [22].

Is it possible to quantify the cost–including disentangling the factors that influence the NNS–of saving a life from the effects of advanced melanoma by secondary prevention? Is it possible to quantify the effects of secondary and tertiary prevention with respect to melanoma mortality rates? Is it possible to predict what might happen to incidence and mortality rates if current melanoma surveillance strategies are maintained, enhanced, or abandoned? Due to the existence of feedback loops and non-linear interactions between various factors, the questions raised above cannot be answered in the affirmative solely by intuition. In contrast, quantitative modelling may yield robust answers. In the spirit of the recommendations of Basu *et al* [23], here I develop a relatively simple–but not too simple–Markov model of melanoma epidemiology based on Australian incidence and mortality data.

I first develop an accurate representation of Australian melanoma incidence and mortality rates over the period 1982–2013, and an accurate representation of projected incidence and mortality rates over the period 2013–2028. Using these models as baseline, the primary aim of the analysis is to determine, both retrospectively and prospectively, quantitative estimates regarding the effects (and costs) of different levels of secondary prevention with respect to incidence and mortality. (By analogy, a climate model may be set up to accurately capture the post-industrialisation rise in average global temperature. By re-running the model without the known increase in atmospheric carbon dioxide levels, it is possible to estimate the impact of this greenhouse gas on global temperature rise). An enhanced level of secondary prevention, for example, would invoke a detection strategy that utilised increased screening frequency and/or the use of additional screening aids, such as full-body photography. Secondary aims include estimating the effects on mortality with variation in the effectiveness of tertiary prevention; to directly compare mortality rates with respect to enhanced secondary versus enhanced tertiary prevention; and finally, to determine the relationship between over-diagnosis levels and melanoma incidence.

The model thus achieves its goals–it can suggest what may happen (or what has happened) given certain scenarios or public health policy decisions. It is likely to be applicable in Western countries where the increased incidence of melanoma over the past three decades mirrors the Australian data. In their summary of the available evidence pertaining to the effectiveness of melanoma screening–prepared for the US Preventative Services Task Force and published in the *Annals of Internal Medicine*–Wolff *et al* write: “*Given the lack of direct evidence*, *modelling studies using available indirect evidence*, *including cost-effectiveness studies*, *may provide some information on the usefulness of screening as a preventative strategy*.” [18].

## Methods

### Markov models

Markov models are a class of deterministic dynamical system models where the next state of the system is uniquely determined by its previous state. A simple Markov model is, for example, a weather model where the states of the system are given by ‘sunny’, ‘rainy’, or ‘cloudy’, and the state of tomorrow’s weather only depends (in a probabilistic manner) on its state today. Markov models are thus fully described by a *transition matrix* that defines the states of the system and the transition probabilities between states. They have been used widely in science; recently Markov models are becoming increasingly popular in modelling biological processes, including cancer evolution [24, 25].

### The model

Can melanoma evolution be considered Markovian? It can: in this case the states of the system are defined according to the stage of disease, while the probability of transitioning to a new state, or remaining in the same state, depends, in a probabilistic manner, only on the current state. Although it is clear that past events can affect the present in cancer evolution, the stochasticity inherent in Markov models allows for heterogeneity, with respect to individuals, in the rates of tumour evolution. Some individuals will thus transition more quickly than others: these individuals, for example, could be considered those with more aggressive disease where past events (such as poor prognosis mutations) can and do influence present events. The model presented here is constructed by first defining the states of the system:

1. **Baseline**
2. **Undiagnosed Stage 1:** invasive melanoma less than 2mm thick
3. **Undiagnosed Stage 2 or 3:** invasive melanoma greater than 2mm thick or melanoma of any thickness with local lymph node involvement
4. **Undiagnosed Stage 4:** distal lymph node and/or organ involvement
5. **Diagnosed (and thus treated) Stage 1:** as above
6. **Diagnosed (and thus treated) Stage 2 or 3:** as above
7. **Diagnosed (and thus treated) Stage 4:** as above
8. **Death**

These states are in accordance with standard staging definitions [26]. The baseline state represents the Australian population at large and can comprise individuals without melanoma, or those with a diagnosis of *in-situ* melanoma (where the latter is defined as Stage 0). Stage 1 is defined as thin invasive melanoma. A novel feature of the model is the introduction of states where the diagnosis has not been made (States 2, 3 and 4 above); in these cases the individual is not aware (or perhaps in denial) that melanoma may be present. Importantly, these individuals will not have had any treatment–for example, excision of the primary lesion in Stage 1 disease–hence their prognoses will differ from their treated counterparts.

### The mean-field approximation

The model is set up as a ‘*mean-field*’ model; that is, the differences between individuals are smoothed out, and averages are used. The model does not take into account details such as age or inherent individual or group risk of progression or death. For example, individuals without melanoma and those with *in-situ* melanoma are grouped together in the baseline cohort. Although they are at different risk for developing undiagnosed Stage 1 disease, averages are used in the model. (Since publicly available melanoma incidence data refer to invasive melanoma, there exists no data on the incidence of *in-situ* melanoma. To include *in-situ* melanoma as a separate state in the model would therefore add inaccuracies and complexity, the latter rendering it less tractable). While the risk death from causes other than melanoma–or the risk of developing melanoma–is higher in a 80 year old individual when compared with a 20 year old individual, and while the risk of developing melanoma in any given year is higher in a red-head with freckles in comparison to an individual of Middle Eastern descent, including such details would greatly increase the number of states and render the model intractable.

### State transitions

**Fig 1:**
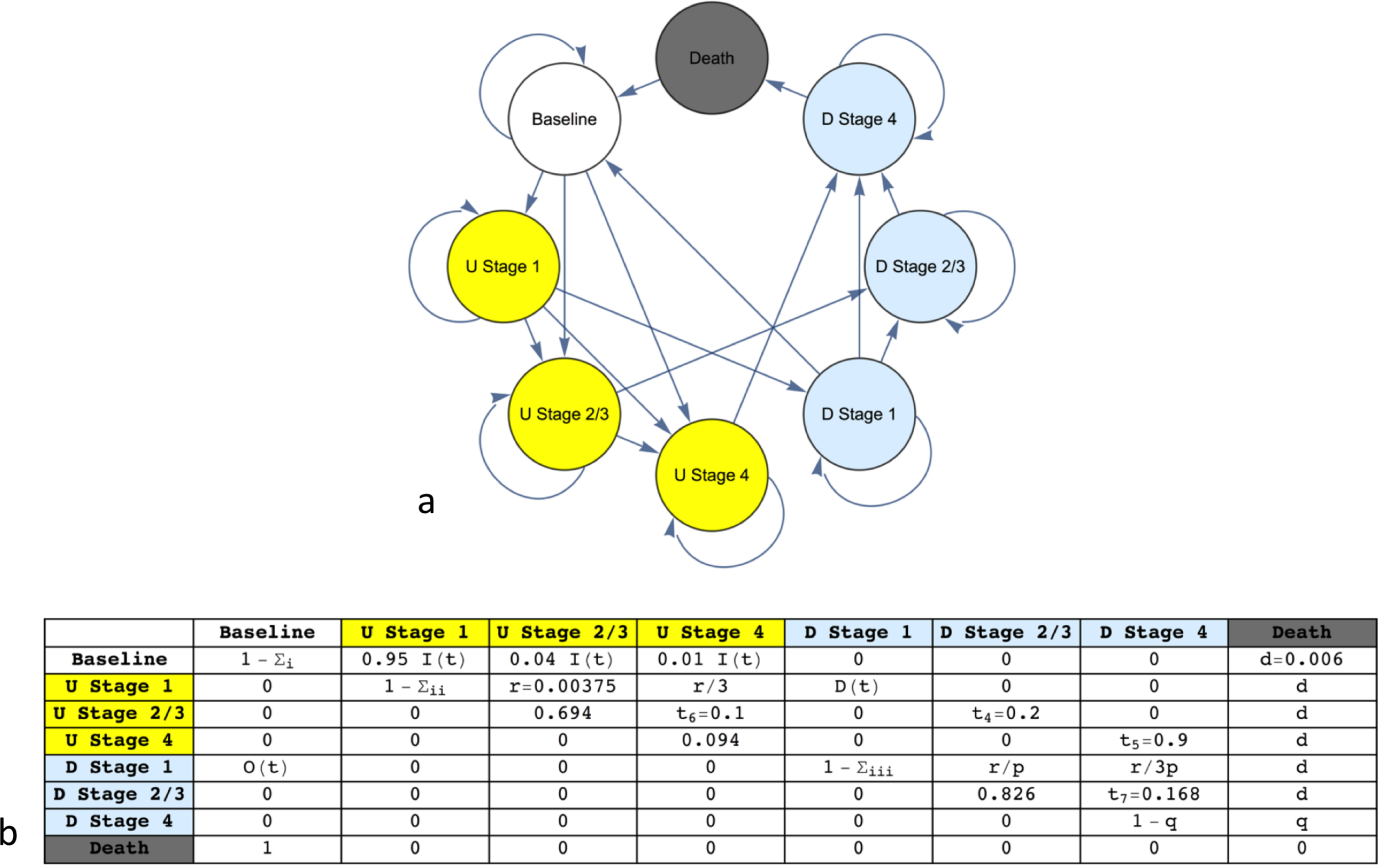
**(a)** State transitions represented by a directed graph. **(b)** The associated transition matrix. In both (a) and (b) undiagnosed (U) and diagnosed (D) disease are shown in yellow and light blue respectively. An iteration of the model corresponds to 1 year. Note the rows sum to 1 since the probability of transitioning to the same state is given by 1 minus the sum of all other entries for that row. The diagonal entry for Stage 1 in yellow thus represents the finite probability that an individual can remain with undiagnosed disease for many years, in keeping with the observation that some patients will not present in a timely manner [28], or their doctor may not make an early diagnosis [29]. This phenomenon increases the likelihood that he or she may transition directly to undiagnosed later-stage disease.

The possible state transitions of the model are shown in Fig 1A as a directed graph while the transition matrix is shown in Fig 1B. Note from Fig 1A that not all transitions are possible (for example, transitioning from diagnosed Stage 4 disease to Baseline) and that it is possible for individuals to skip Stage 2/3 (for example, transitioning from diagnosed Stage 1 to diagnosed Stage 4 –this corresponds to a patient who has had a thin invasive melanoma excised, but re-presents with metastatic disease). Note that there are no transitions from baseline to diagnosed invasive disease. In general, this feature accounts for the arbitrarily short but non-zero time patients may exist in the invasive but undiagnosed state. The important features of the transition matrix (Fig 1B) are (i) in each row the probabilities sum to 1; (ii) the model comprises eleven parameters (transition probabilities); (iii) three of those probabilities are time-dependent; (iv) the cycle time is one year; (v) the transitions from death to Baseline occur with probability 1 and act to keep the population constant; and (vi) death can occur at any stage with low probability from causes other than melanoma (these latter transitions have been omitted from the graph in Fig 1A to avoid clutter).

### Transition probabilities

Usually the probabilities in the transition matrix of a Markov model are invariant in time; however, in the following I introduce a *dynamic* model–in this case some of the probabilities of the transition matrix can change continuously in time. In contrast to models with fixed transition probabilities, dynamic models will never reach their stationary states; they will be out-of-equilibrium dynamical systems.

The values and their ranges (if applicable) of all eleven probabilities are shown in Table 1. There are three time-dependent parameters present, denoted ***I****(t)*, ***D****(t)* and ***O****(t)* (see Fig 1B). ***I****(t)* denotes the probability, per year, of developing (undiagnosed) Stage 1, Stage 2/3 or Stage 4 disease given the baseline state, where ***I****(t)* is partitioned among the three transitions depending on their respective likelihoods. Note from Fig 1B that the transition to undiagnosed Stage 1 is overwhelmingly more likely than a direct transition to Stage 4. Yet the latter can occur, albeit with low probability (for example, when an individual with previously known or unknown *in-situ* melanoma or leptomeningeal melanosis develops metastatic melanoma). ***D****(t)* denotes the probability, per year, of having a diagnosis made of Stage 1 disease given the presence of undiagnosed Stage 1 disease. The value of ***D*** thus quantifies the level of secondary prevention. Larger values correspond to an enhanced detection strategy (explained in the Introduction) and thus a greater probability of diagnosis of Stage 1 disease given undiagnosed Stage 1 disease. Finally, ***O****(t)* denotes the probability of having a diagnosis of Stage 1 disease but immediately transitioning to the baseline state. ***O****(t)* thus represents the concept of over-diagnosis, where melanoma is diagnosed but the lesion has no life-threatening potential.

**Table 1:** The eleven parameters of the model.

	Description	Value(s)	Justification	Ref.
***I(t)***	Probability of developing undiagnosed invasive melanoma	0.00031–0.00052	Fitted to data	AIHW*, 2016
***D(t)***	Probability of diagnosis of Stage 1 given undiagnosed Stage 1	0.01–0.10	Delay in diagnosis data	Ref. 28
***O(t)***	Probability of overdiagnosis given Stage 1 diagnosis	0.00–0.10	Plausible range	Ref. 21
***r***	Probability of undiagnosed Stage 1 progressing to undiagnosed Stage 2/3	0.00375	Fitted to data	
***p***	Ratio of the probability of undiagnosed versus diagnosed progression of Stage1 to later-Stage	2, 5, 15, 50, 125	Plausible range	
***q***	Probability of death given Stage 4	0.185, 0.23, 0.30, 0.37	Fitted to data; 5 year survival data	Ref. 29
***d***	Probability of death from causes other than melanoma	0.006	Fitted to whole- population data	AIHW*, 2016
***t***_***4***_	Probability of progression from undiagnosed Stage 2/3 to diagnosed Stage 2/3	0.2	Plausible value; value not critical	
***t***_***5***_	Probability of progression from undiagnosed Stage 4 to diagnosed Stage 4	0.9	Plausible value; value not critical	
***t***_***6***_	Probability of progression from undiagnosed Stage 2/3 to undiagnosed Stage 4	0.1	Plausible value; Value not critical	
***t***_***7***_	Probability of progression from diagnosed Stage 2/3 to diagnosed Stage 4	0.168	Fitted to data; 5 year survival data	MIA**, 2016

*Australian Institute for Health and Welfare [15].

** Melanoma Institute Australia [26].

Note the functions ***I***, ***D*** and ***O*** are time-dependent variables. The values of ***I****(t)* are determined such that the resultant model accurately fits the AIHW data. The ‘Value(s)’ column reveals the continuous range of values for the time-dependent variables, and the discrete (either single or multiple) values for the other parameters. All probabilities (values) are per person per year.

Why are the aforementioned parameters chosen as time-dependent, while the others are fixed? ***I****(t)*, which I call the *intrinsic incidence*, must increase such that the *diagnostic incidence–*which is a property of the model’s output–increases. Indeed, the diagnostic incidence of melanoma in Australia, reported at the AIHW website, has increased over the period 1982–2013 [15]. ***D****(t)* has also increased over the period 1982–2013; this reflects the effects of public health campaigns on the behaviour medical practitioners and the public with respect to diagnostic vigilance. Finally, ***O****(t)*, the probability of over-diagnosis, is a time-dependent variable since over-diagnosis rates are thought to have significantly increased over the past few decades [21].

An important parameter is the ratio of the probability of progression of undiagnosed and diagnosed Stage 1 disease to later-stage disease, denoted by ***p*,** and given by ***r/(r/p)***, where ***r*** is the probability of progression, per year, of transitioning from undiagnosed Stage 1 disease to undiagnosed Stage 2/3 disease, and ***r/p*** is the probability of progression, per year, of transitioning from diagnosed Stage 1 disease to diagnosed Stage 2/3 disease. Note from Fig 1B and Table 1 that ***r/3*** is the probability of transitioning from undiagnosed Stage 1 disease to undiagnosed Stage 4 disease, while ***r/3p*** is the probability of transitioning from diagnosed Stage 1 disease to diagnosed Stage 4 disease. The latter observation reflects the plausible assumption that transitioning to Stage 2/3 is more likely than transitioning directly to stage 4, irrespective of whether Stage 1 disease is undiagnosed or diagnosed. The value of ***p*** thus quantifies the decreased likelihood of progression of Stage 1 disease with treatment, where the latter is usually a wide local excision. All probabilities of progression from Stage 1 disease (both undiagnosed and diagnosed) are thus determined by the values of ***r*** and ***p***.

The parameter ***q*** represents the probability of transitioning, per year, from diagnosed Stage 4 disease to death. Although ***p*** and ***q*** are not defined as time-dependent functions, various plausible values are used for each in the analysis, described below. The parameter ***d***, which is fixed throughout the analysis, represents the probability, per year, of transitioning from any state to death, where the cause of death is not melanoma. Likewise, the parameters ***t***_***4***_ ***–t***_***7***_ are fixed throughout the analysis and are defined in Table 1.

### Methodology

The Results section is divided into four parts. In the first part I investigate the state space of models for two values of ***D****(0)* (the value of ***D*** in 1982) and for five values of ***p***. For each of ten models, I determine conditions on the probabilities that yield an accurate steady state distribution of state frequencies such that the diagnostic incidence rate and mortality rate match their respective reported values in 1982. I then determine specific curves ***I****(t)* and ***D****(t)* such that the model incidence and mortality rates closely match the reported data over the period 1982–2013. How is the latter achieved? For each of ten potential models, I define three categories of monotonically increasing curves ***D****(t)* ranging from *t = 0* to *t = 31* that reflect the increasing detection likelihood of Stage 1 disease over the period 1982 to 2013. I choose a parabolic concave-up curve, parabolic concave-down curve, a sigmoid curve and a straight line as representative of the possible trajectories. For each category of curve I choose different values of ***D****(31*) (the value of ***D*** in 2013), each representing a different maximal increase. For any ***D****(t)* I then perform a detailed search through the parameter space of parabolic curves ***I****(t)*, finding a suitable ***I****(t)* such that the simulated Markov model best fits the incidence and mortality data over the period 1982–2013. (For curves of the type ***I****(t)* = ***a + b****t* ***+ c****t*^*2*^ where ***a*** is known (baseline) I search through over 10,000 combinations of values for ***b*** and ***c***, where each parameter spans two orders of magnitude and is partitioned 100-fold. The extreme values of ***b*** and ***c*** generate increasingly erroneous models. To minimize the problem of overfitting, I avoid using polynomials of degree 3 or above). The resultant ten models are best-fit models with respect to the state space of the two chosen values of ***D****(0)* and the five chosen values of ***p***.

In the second part I use the model to investigate the years 1982–2013 with respect to variation in ***D****(t)*. I disentangle the effects of enhanced secondary prevention with respect to Stage 1 disease, represented by an increasing ***D****(t)*, in comparison with no increase to ***D****(t)*.

In the third part I use the model to investigate the years 1982–2013 with respect to the mortality effects of hypothetical improvements in the efficacy of treatment of Stage 4 disease (tertiary prevention) by reducing the value ***q***, and I investigate the effects of different time-dependent increased levels of over-diagnosis, denoted ***O****(t)*, with respect to incidence rates.

In the fourth part I extend the Markov model by approximating the projected melanoma incidence and mortality, given by Whiteman *et al*, from 2013 to 2028 [27]. I then investigate the future effects on incidence and mortality by either varying ***D****(t)* or by reducing the value of ***q*** from baseline.

## Results

### Replicating the AIHW data from 1982–2013

For the ten models, the values of ***D****(0)* are given by 0.01 and 0.05, while the values of ***p*** are given by 2, 5, 15, 50, and 125. With respect to ***D****(0)*, values of 0.01 and 0.05 correspond to a one in one hundred and a one in twenty chance, respectively, per year, of having a diagnosis made of Stage 1 disease, given the presence of undiagnosed Stage 1 disease. Although the exact value of ***D*** in 1982 is unknown, it is unlikely to be larger than 0.05. Indeed, a large study reported by Richard *et al* reveals long delays among individuals in recognising a melanocytic lesion as suspicious (greater than 5 years in 37% of the cohort), and further long delays in seeking medical attention (greater than 1 year in 12% of the cohort) [28]. In other cases, delays may be due the treating physician not making a timely diagnosis [29]. While the value of ***p*** is unknown, it is unlikely to be less than 2 or greater than 125.

I now describe one of the ten models in detail; this model is characterised by ***D****(0)* = 0.05 and ***p*** = 15. As noted above, the former value corresponds to a one in twenty probability, in 1982, of having Stage 1 melanoma diagnosed given the presence of undiagnosed Stage 1 disease. The latter value corresponds to a 15-fold increased likelihood that undiagnosed Stage 1 disease will progress in any year in comparison with diagnosed (and thus treated) Stage 1 disease.

**Fig 2:**
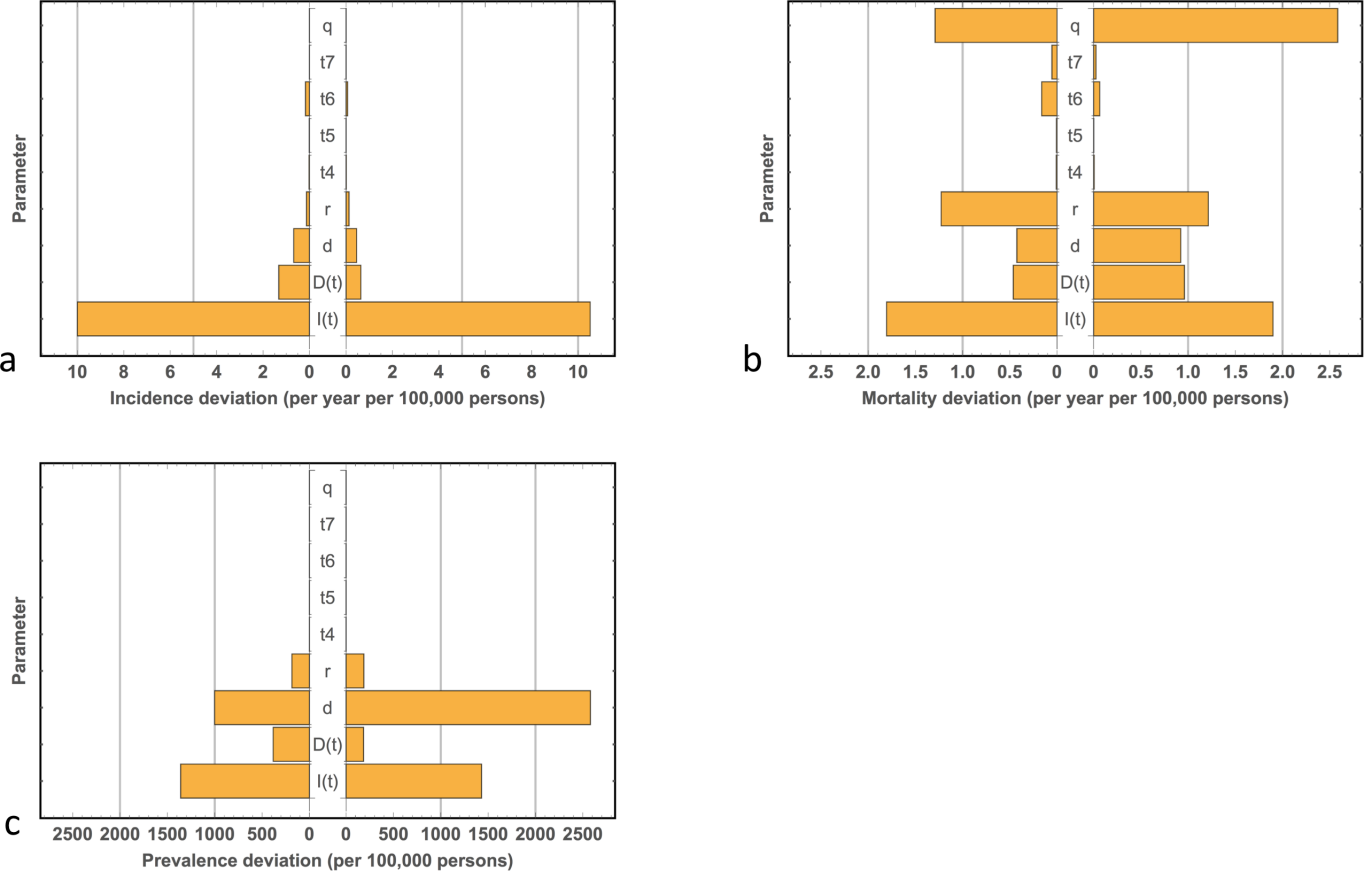
Monte-Carlo sensitivity analysis. **(a)** Deviations in melanoma incidence. **(b)**, Deviations in melanoma mortality. **(c)** Deviations in the prevalence of diagnosed thin melanoma. All deviations are calculated with respect to the model parameters. For a given parameter, the model was run to steady state for each of 10,000 normally distributed random values about its 1982 steady state value (with a standard deviation of 10% of the steady state value) while keeping all other parameters fixed at the values that yield the 1982 steady state (where zero deviation is given by an incidence of 27.08 per year per 100,000 persons, a mortality of 4.76 per year per 100,000 persons and the prevalence of diagnosed thin melanoma of 3215 per 100,000 persons). Note how the majority of variability within the model is due to variation in the time-dependent variables ***I****(t)* and ***D****(t)*, with the exception of the value of ***q*** which has a disproportionate influence on mortality.

Running the model to steady state to replicate the initial (1982) incidence and mortality data reveals that some of the parameters are constrained, while others are less constrained. This finding is captured by the Monte-Carlo sensitivity analysis, corresponding to the year 1982, shown in Fig 2. Note the majority of sensitivity within the model lies with the time-dependent parameters ***I****(t)* and ***D****(t)*. These functions require fine-tuning to allow the model to yield accurate output. Likewise, the value of ***d*** is fine tuned such that the model output matches whole-population prevalence and mortality rates (see below). The value of ***r*** has a moderate effect on mortality; its value thus requires fine-tuning to allow the model to replicate mortality rates. Finally, the value of ***q*** has a marked effect on mortality rates but no effect on incidence rates. Its value, determined by a fit to the data, is given by 0.37, a value that corresponds to a one in three chance of death from Stage 4 disease per year. Reassuringly, this value of ***q*** corresponds to a five-year survival of 12%, a value in close agreement with published data. (Sandru *et al* report on a five-year survival for Stage 4 disease between 5–19% [30]). Finally, the values of the transition probabilities ***t***_***4***_ ***–t***_***7***_ exhibit very little sensitivity with respect to the model’s output. While care is taken in choosing plausible values, inaccuracies will not therefore compromise the integrity of the model.

**Fig 3:**
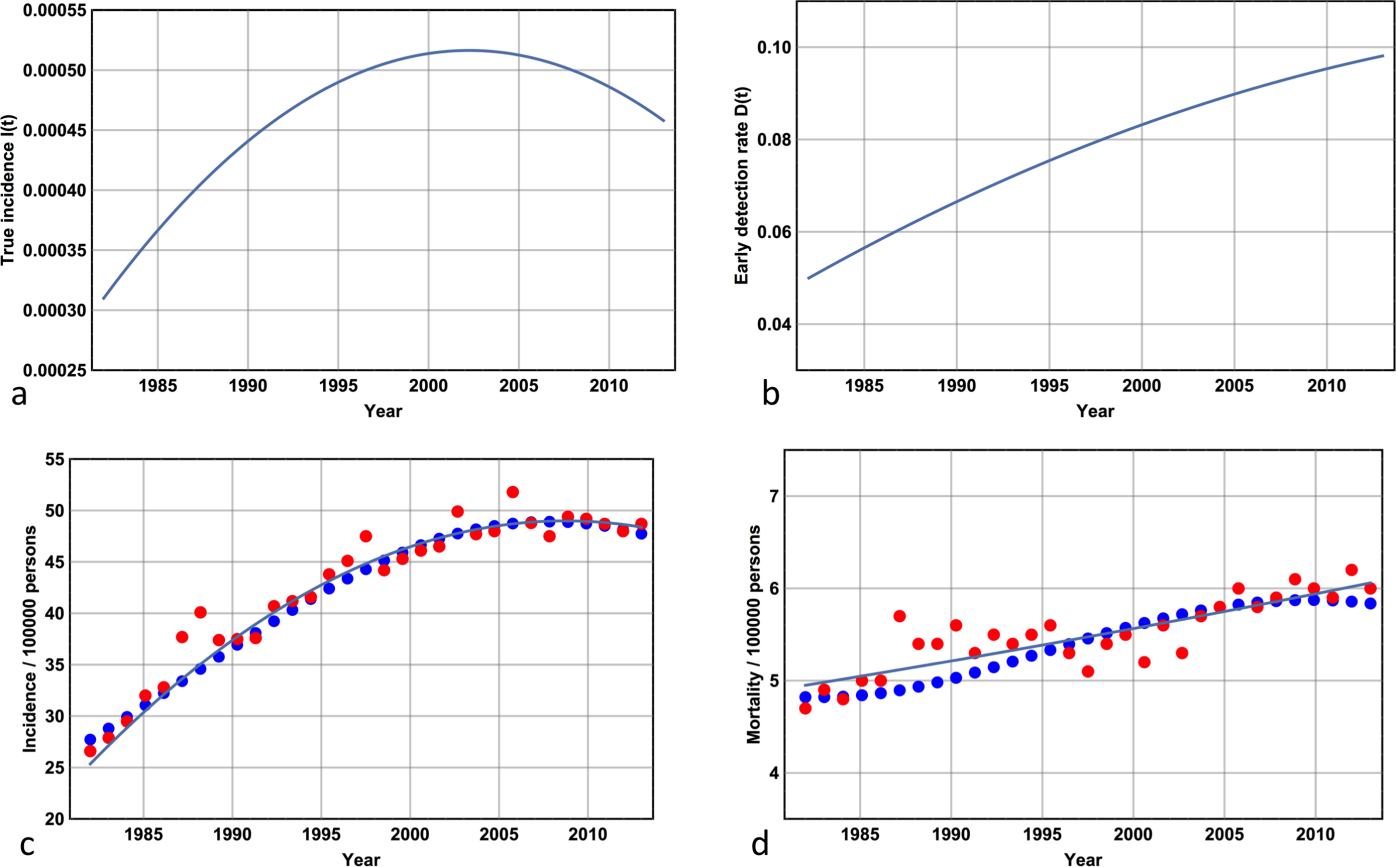
The Markov model for *D*(0) = 0.05 and *p* = 15. **(a)** The true incidence curve ***I****(t) = 0*.*00031 + 0*.*00068t – 0*.*00056t*^*2*^ *(t is rescaled)* for the period 1982–2013. Note how this curve peaks around 2003. **(b)** The detection likelihood, ***D****(t) = 0*.*05 + 0*.*075t* – *0*.*075t*^*2*^ *(t is rescaled)* corresponding to the probability of diagnosis of Stage 1 melanoma per year in an individual with hitherto undetected Stage 1 melanoma. Note the incremental increase over the period 1982–2013. **(c)** Actual incidence data (orange dots), least-squares curve of best fit to these data (continuous line), and the Markov approximation (blue dots) over the period 1982–2013. Note the accuracy of the Markov approximation, and note how this curve peaks later than the true incidence curve given by (a). **(d)** Mortality data with the same interpretation as (c). Note the increase in mortality from ~5 persons per year per 100,000 persons in 1982 to ~6 persons per year per 100,000 persons in 2013. (All actual incidence and mortality data are reported at the Australian Institute of Health and Welfare website [15]).

I now run the Markov model to 2013, where a single iteration corresponds to one year. The best fit curves for ***I****(t)* and ***D****(t)* are shown in Fig 3A and 3B respectively, while the Markov approximation is shown in Fig 3C and 3D. Note that ***D****(31)* is given by 0.10 –this corresponds to a one in ten chance, in 2013, of having a diagnosis of Stage 1 disease made given the presence of undiagnosed Stage 1 disease; its value equates to a doubling of this likelihood since 1982. Note that ***I****(t)* peaks around 2003 –this precedes the diagnostic incidence peak shown in Fig 3C by 5 years and demonstrates that the true incidence has reached a plateau and possibly been in decline for over a decade (as of 2016). Note the accuracy of the Markov approximation: simulated values accurately track the least squares approximation to the reported data throughout the period 1982–2013 with an error rate less than 4.5% (Fig 3C and 3D). The state frequencies are also accurate. As of 2010 the number of people living with melanoma diagnosed after 2005 is given by 48,937, while the mortality rate from any cause is documented at ~580 persons per 100,000 per year [15]. These data compare favourably with the model results for ***D****(0)* = 0.05 and ***p*** = 15 in 2010 (89,500 and 602 per 100,000 persons respectively). The value of the state frequency of state 5 as of 2010 will take into account all individuals diagnosed with thin invasive melanoma from 1982 with the exception of those that have progressed or those that have died from any cause. Hence roughly half of all people living with diagnosed and treated thin invasive melanoma as of 2010 were diagnosed prior to 2005.

### Varying the detection probability of Stage 1 melanoma for the years 1982–2013

**Fig 4:**
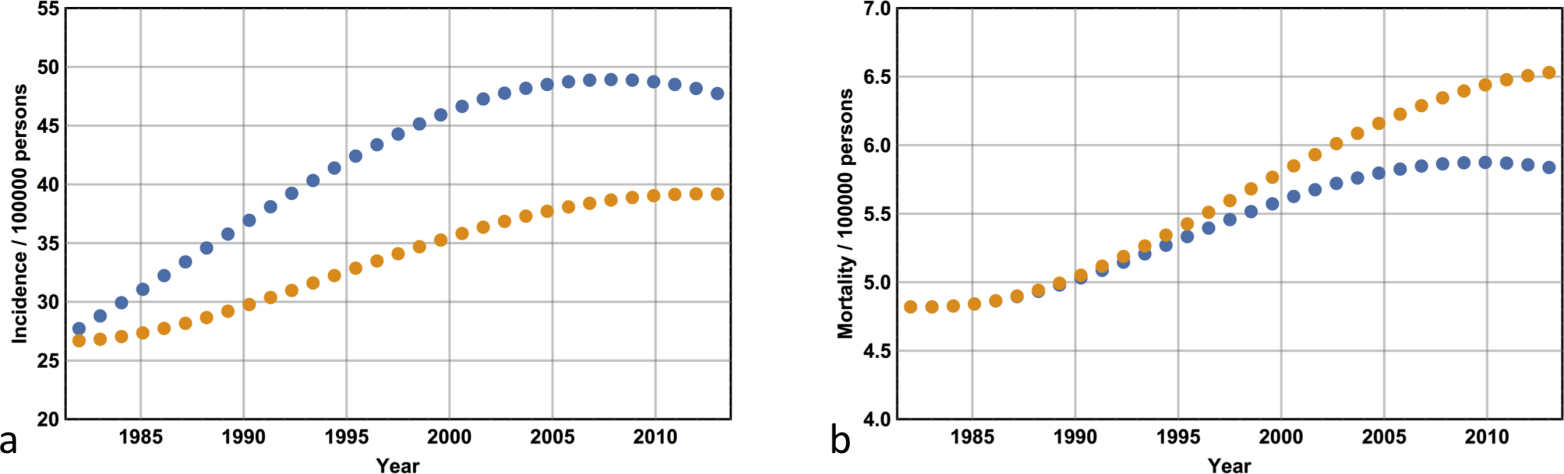
The effects of enhanced secondary prevention for the years 1982–2013. **(a)** Diagnostic incidence curves. **(b)** Mortality curves. For both plots, enhanced secondary prevention is represented by the blue dots while secondary prevention that remains at 1982 levels is represented by the orange dots. Note how a lack of enhanced secondary prevention results in a lower diagnostic incidence rate but an increased and divergent mortality rate.

By keeping ***D****(t)* fixed at its initial value of 0.05 for the duration *t* = 0 to *t* = 31 I now simulate the years 1982–2013 without an increase in the detection probability of thin invasive melanoma (Fig 4A and 4B). Clearly, not increasing the detection likelihood of Stage 1 disease has two consequences: first, it results in a much lower diagnostic incidence rate; and second, it results in a higher mortality rate. While a significant fraction of the ‘alarming’ melanoma diagnostic incidence rate increase over the past 30 years is simply due to earlier detection, it better reflects the increase in the true incidence ***I****(t)* (Fig 3A). Table 2 shows, for all 10 models, the quantitative results associated with a monotonically increasing ***D****(t)* in comparison with a ***D****(t)* that remains at baseline. Note the robustness of the model with respect to the two values of ***D****(0)* and the five values of ***p*** and note how the cost (estimated by calculating the ratio of melanoma excisions to lives saved) of saving one life from melanoma is larger for ***D****(0)* = 0.01 in comparison with ***D****(0)* = 0.05 and varies inversely with ***p*** for any ***D****(0)*. Thus the lowest cost of saving a life from melanoma occurs when ***D****(0)* = 0.05 and ***p*** = 125. This corresponds to the situation where the early detection likelihood is high, and the stakes for doing so are high. In practice, the exact values of ***D****(t)* and ***p*** are unknown; however, by interpolation, I can estimate the number of early invasive melanoma excisions over the period 1982–2013 required to save one life to be within the range 50 to 120.

**Table 2:** Effects associated with enhanced secondary prevention for the years 1982–2013.

*D(0)*	*p*	*Error*	*TE*	*TM*	*EE*	*RM*	*Ratio*
**0.01**	**2**	0.039	1321	167	434	0.6	654
**0.01**	**5**	0.043	1335	166	430	1.6	269
**0.01**	**15**	0.042	1328	166	438	2.5	173
**0.01**	**50**	0.041	1328	167	435	2.9	149
**0.01**	**125**	0.043	1335	166	438	2.9	148
**0.05**	**2**	0.030	1310	168	308	1.2	251
**0.05**	**5**	0.036	1321	165	304	4.1	74
**0.05**	**15**	0.036	1297	167	255	6.7	37
**0.05**	**50**	0.037	1294	168	248	9.4	26
**0.05**	**125**	0.037	1293	169	244	10.5	23

This Table corresponds to the simulations shown in Fig 4. Results for all 10 models (***p*** = 2, 5, 15, 50 and 125) and ***D****(0)* = 0.01 (top 5 rows) and ***D****(0)* = 0.05 (bottom 5 rows). Note the accuracy of the Markov approximation to the AIHW data (*error* column) reflected in the *total number of excisions* (*TE*) and the *total mortality* (*TM*). For comparison, the total number of diagnoses from the Australian Institute of Health and Welfare for the period 1982–2013 is 1319, while the total mortality over the same period is 171. The *excess number of excisions* due to an increased probability of diagnosis of thin invasive melanoma given the presence of undiagnosed thin invasive melanoma (compared with keeping the probability of diagnosis of thin invasive melanoma at 1982 levels until 2013) is shown (*EE*), while the *reduced mortality* due to increased probability of diagnosis of thin invasive melanoma is shown (*RM*). Finally, the ratio of *EE* to *RM* is shown (*Ratio*). Note that, for example, when ***D****(0)* = 0.05 and ***p*** = 15, enhanced secondary prevention for the years 1982–2013 has led to 255 extra excisions but 6.7 less deaths. All figures are per 100,000 persons.

### Varying the likelihood of death from Stage 4 disease and investigating the effects of over-diagnosis for the years 1982–2013

**Fig 5:**
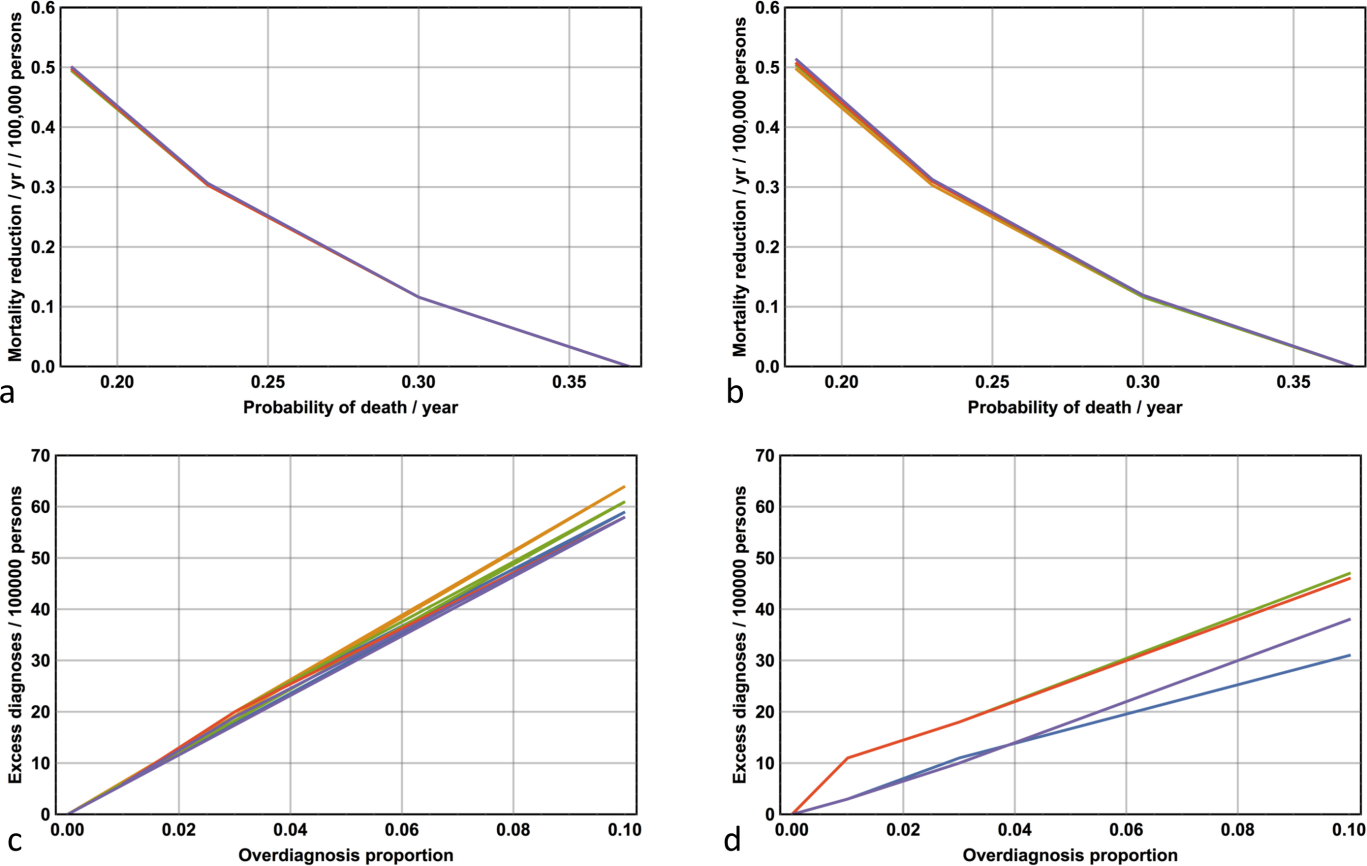
The effects of improved efficacy associated with treating late-stage disease and the effects of over-diagnosis for the years 1982–2013. **(a)** Average mortality reductions per year per 100,000 persons as a function of ***q*** for all ***p*** where ***D****(0)* = 0.01 and **(b) *D****(0)* = 0.05. Note the robustness of results with respect to both ***p*** and ***D****(t)* (all five plots for different values of ***p*** are closely superimposed). **(c)** Excess diagnoses per 100,000 persons as a function of maximal over-diagnosis proportion for all ***p*** where ***D****(0)* = 0.01 and **(d) *D****(0)* = 0.05.

How else can lives be saved in melanoma? One possibility is to increase the efficacy in the treatment of late stage disease. This effect can be implemented in the model by varying the parameter ***q***, the probability of death per year given the presence of Stage 4 disease. The baseline probability is given by ***q*** = 0.37, and I investigate the effects on mortality rates for the years 1982–2013 with the reduced values ***q*** = 0.3, ***q*** = 0.23 and ***q*** = 0.185. The results are shown in Fig 5A and 5B. Note the robustness of the results with respect to variation in ***D****(0)* and ***p***. Note if the value of ***q*** is reduced to 0.185, corresponding to a 50% reduction in the likelihood of death from Stage 4 disease (per year), the average mortality rate drops by 0.5 per 100,000 persons per year.

I now provide some quantitative estimates on the effects of over-diagnosis on incidence rates. Fig 5C and 5D show, for all 10 models, the number of excess diagnoses per 100,000 persons over the period 1982–2013 given a monotonically and linearly increasing over-diagnosis rate, given by ***O****(t)*, where ***O****(0)* = 0 and ***O****(31)* is set to a probability equal to 1%, 3% or 10% of the probability associated with a diagnosis of Stage 1 disease. Note that, for example, if one in thirty Stage 1 melanomas were over-diagnosed in 2013 then 10–20 excess diagnoses per 100,000 persons over the period 1982–2013 would have occurred.

### Projections for the years 2014–2028

**Fig 6:**
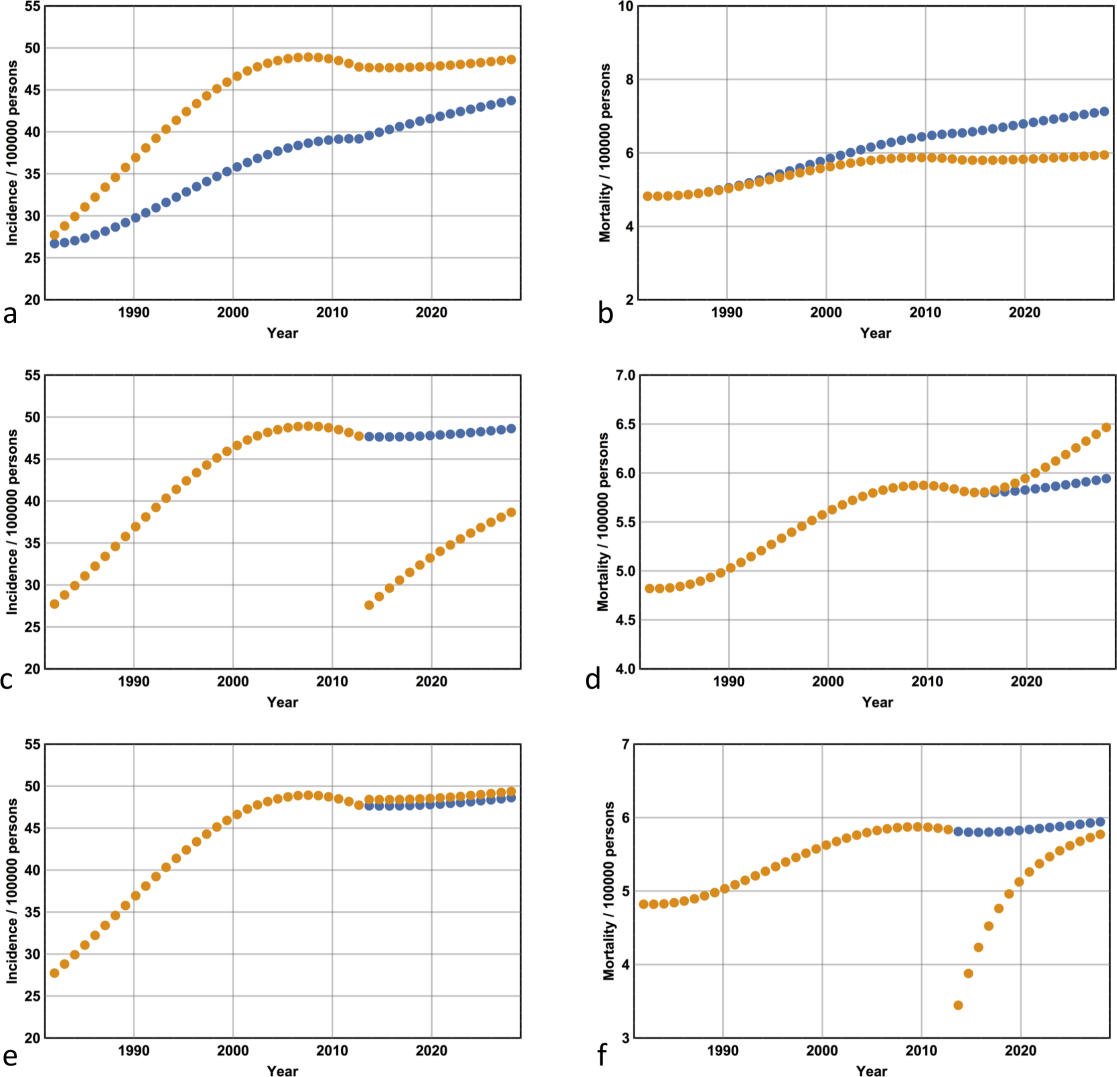
Expected melanoma incidence and mortality for the years 2013–2028. **(a)** Extending enhanced secondary prevention at its 2013 level to 2028 (where incidence is given by the orange dots) compared with a continuation of 1982 levels (blue dots) reveals a converging diagnostic incidence rate and **(b)** a divergent mortality rate. **(c)** Reverting to the 1982 detection probabilities of Stage I melanoma in 2013 results in a sharp drop in diagnostic incidence that rebounds rapidly, and **(d)** a divergent mortality curve. **(e)** Reducing the risk of death from late-stage disease in any year by half in 2013 results in no change to incidence and **(f)** a sharp drop in mortality that quickly rebounds to baseline levels.

I now extend the Markov model out to 2028 by approximating the incidence and mortality projections of Whiteman *et al* [27]. I consider three scenarios: first, the situation where I compare the incidence and mortality associated with a continuation of the scenario presented in the previous section. I compare the incidence and mortality obtained from a ***D****(t)* that remains fixed at its 2013 level with the incidence and mortality associated with a value of ***D****(t)* that remains fixed at its 1982 level (which has not changed by 2013). Fig 6A and 6B show the case where ***D****(0)* = 0.05 and ***p*** = 15, while for all ten models; these data are shown in Table 3. Note from Fig 6A and 6B that by keeping ***D****(t)* at 0.05 –its 1982 level–melanoma incidence remains lower and the mortality increases in comparison with the ‘business as usual’ approach. By keeping ***D****(t)* at its 2013 level with the incidence and mortality associated with a value of ***D****(t)* that remains fixed at its 1982 level (which has not changed by 2013), the gains made from the period 1982–2013 are amplified for the years 2013–2028: mortality has decreased dramatically, and the ratio of melanomas excised to lives saved is significantly reduced (compare Tables 2 and 3).

**Table 3:** Expected effects of continuing with enhanced secondary prevention for the years 2013–2028.

*D(0)*	*p*	*TE*	*TM*	*EE*	*RM*	*Ratio*
**0.01**	**2**	750	85.7	283	1.9	148
**0.01**	**5**	783	87.2	292	4.3	67
**0.01**	**15**	769	85.0	290	6.6	43
**0.01**	**50**	769	85.0	288	7.6	37
**0.01**	**125**	792	87.7	297	7.7	38
**0.05**	**2**	727	92.0	113	2.9	38
**0.05**	**5**	732	88.7	109	8.8	12
**0.05**	**15**	719	87.7	93	15.5	6
**0.05**	**50**	719	87.0	89	20.4	4
**0.05**	**125**	712	86.2	84	22.1	3

This Table corresponds to the simulations shown in Fig 6A and 6B, thus the comparison is between secondary prevention strategies maintained at 2013 levels for the years 2013–2028 versus a continuation of secondary prevention strategies that persist at 1982 levels. Shown are the projected results, interpreted identically to Table 2. In comparison with the period 1982–2013 (Table 3) note that the ratios of *EE* to *RM* are less. Thus if enhanced secondary prevention is extended beyond 2013 the advantages (compared with leaving secondary prevention at 1982 levels) become more significant since fewer excisions are required to save lives.

Second, I consider the situation where ***D****(t)* follows its normal trajectory from 1982 to 2013 but is then re-adjusted to its 1982 value and remains fixed at that value until 2028. This scenario is compared with the situation where ***D****(t)* is held at its 2013 level until 2028. The former corresponds to reverting back to 1982 secondary prevention levels. The results are shown in Table 4 for all ten models. Representative plots for the years 1982–2028 are shown for ***D****(0)* = 0.05 and ***p*** = 15 (Fig 6C and 6D). Note that from 2013 onwards the incidence rate initially falls dramatically but subsequently climbs rapidly, while the mortality rate increases. These effects run counter to the results shown in Table 3: in comparison with the latter, the number of lives saved is reduced, and the fraction of melanomas excised to lives saved has increased.

**Table 4:** Expected effects of abolishing increased melanoma surveillance in 2013.

*D(0)*	*p*	*TE*	*TM*	*RE*	*EM*	*Ratio*
**0.01**	**2**	411	85.9	339	0.2	1695
**0.01**	**5**	435	87.9	348	0.7	497
**0.01**	**15**	420	86.0	349	1.0	349
**0.01**	**50**	422	86.3	347	1.3	266
**0.01**	**125**	429	87.4	363	-0.3	N/A
**0.05**	**2**	473	92.4	254	0.4	635
**0.05**	**5**	481	90.4	251	1.7	107
**0.05**	**15**	505	90.7	214	3.0	71
**0.05**	**50**	509	91.3	210	4.3	48
**0.05**	**125**	507	91.0	205	4.8	42

This Table corresponds to the simulations shown in Fig 6C and 6D. Projected model results for the years 2013–2028 where enhanced secondary prevention is abandoned in 2013, versus a continuation of 2013 secondary prevention levels. Abandoning secondary prevention in 2013 results in a significant number of reduced excisions (*RE*) but an excess mortality (*EM*).

**Fig 7:**
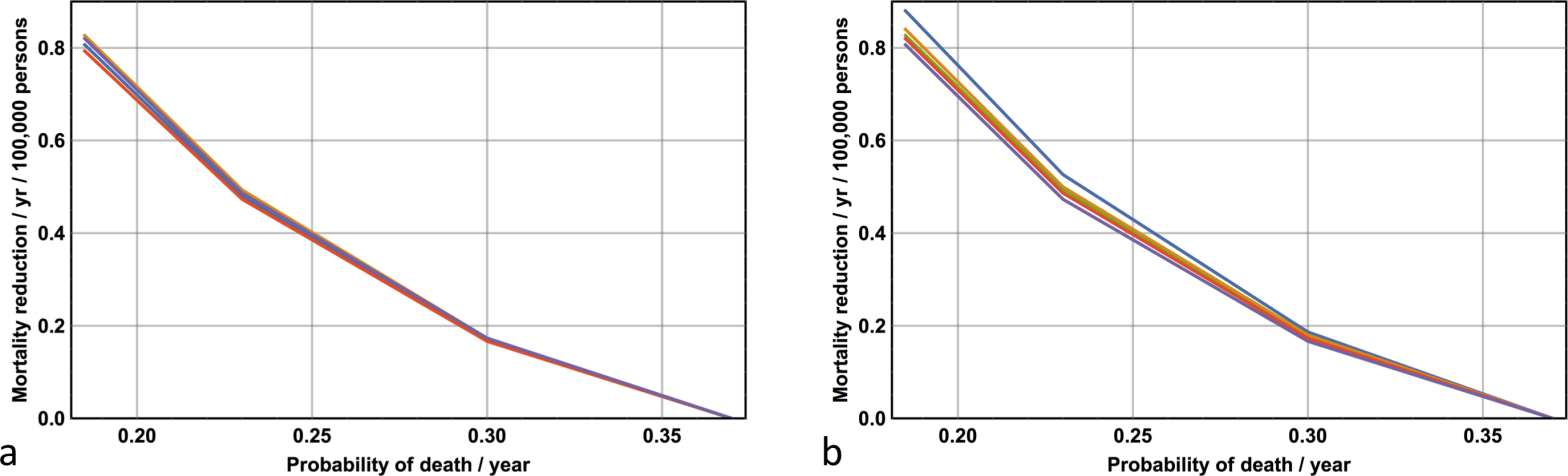
The expected effects associated with improved efficacy in treating late-stage disease for the years 2013–2028. **(a) *D****(0)* = 0.01. **(b) *D****(0)* = 0.05. Projected reductions in mortality (per year per 100,000 persons) as a consequence of increased likelihood of surviving the effects of late-stage disease for all values of ***p***. Note the robustness of results for all 10 models (all the lines representing different values of ***p*** are nearly superimposed).

Third, I consider the situation where ***D****(t)* is maintained at its 2013 level but ***q*** is lowered in 2013 and kept at that value until 2028. This scenario corresponds to improvements in the efficacy of treatment of late stage disease. Data for all ten models with respect to four values of ***q*** (0.37, 0.30, 0.23, 0.185) are shown in Fig 7, while representative plots for ***q*** = 0.185, ***D****(0)* = 0.05 and ***p*** = 15 are shown in Fig 6E and 6F. Note from Fig 6F how the mortality rate initially drops but subsequently climbs rapidly, and from Fig 6E that the incidence remains unchanged.

## Discussion

The results presented above help clarify the relationship between true incidence, diagnostic incidence, and early detection likelihoods. I have shown that the recent slowing of the diagnostic incidence in Australia is due to a peaking of the *true* incidence at around 2004. There is a delay of about 5 years between the peaking of the true incidence curve and the diagnostic incidence curve, reflecting the delay between the onset of undetected melanoma, and the diagnosis of Stage 1 melanoma. Note, however, that this does not mean there is a delay of 5 years in diagnosis for every individual who develops melanoma. Shih *et al* reported on the expected incidence rates of melanoma for the years 1988–2004 without the effects of ‘*Slip*, *Slop Slap*’ and ‘*SunSmart*’, where the latter began in 1988 [2]. In contrast to the results presented in Fig 4A, incidence rates were expected to be *higher* had the public health campaign been non-existent. What is the cause of this discrepancy? Shih *et al* did not disentangle the concept of true incidence and diagnostic incidence, and did not take into account the effects of enhanced secondary prevention with respect to diagnostic incidence. If the public health campaigns, via primary prevention, reduced true melanoma incidence for the years 1982 onwards, then I could have explicitly included an absence of primary prevention in the model by suitably upwardly adjusting the true incidence curve, given by Fig 3A. However, there would be significant guesswork in estimating such a curve; for example, would the peak observed around 2004 disappear? Yet any rise in true incidence will still be offset by the reduction in diagnostic incidence due to decreased surveillance such that the overall diagnostic incidence, over the years 1982–2013, would still be unlikely to be greater than baseline.

Why is the incidence, per 100,000 persons, of melanoma in Australia still not increasing? While the incidence of melanoma is expected to continue to rise in individuals over 80 years of age, it is expected to remain stable in the 60 to 79 year-old age group, and fall in individuals less than 60 years of age [27]. Since melanoma is much more common in the elderly these opposing effects balance–incidence projections suggest a stable incidence rate overall for the next 15 years [27]. However, despite a stable incidence rate, the absolute numbers of new diagnoses are expected to increase due the combined effects of an aging population and population growth [27].

Three explanations for the expected decline in incidence in cohorts less than 60 years of age have been postulated: first, a reduction in ambient ultraviolet light exposure from an early age as a consequence of the introduction of the aforementioned public health campaigns in the early 1980s; second, changes in behaviour leading to less ambient ultraviolet light exposure–such as more time indoors on computers–not attributable to the public awareness campaigns *per se* [27]; and third, a changing demographic in Australia where the recent immigration of young people not susceptible to melanoma has led to skewed data [31]. While the consensus view is that the first explanation is likely to be the most significant, the relative contributions of each, particularly the latter, are immaterial with respect to our model given its mean-field nature.

The second effect of the introduction of the public awareness campaigns that began in the early 1980s is to increase the detection likelihood of Stage 1 melanoma–an effect that I have shown significantly increases diagnostic incidence rates. Thus, as I noted earlier, part of the ‘alarming’ rise in diagnostic melanoma incidence between 1982 and 2013 is simply due to earlier detection. Has the increase in Stage 1 melanoma diagnoses improved health outcomes? The answer is yes–I have shown that mortality for the years 1982–2013 is reduced when early detection increases in comparison with mortality rates associated with no increase to early detection. But there is a trade-off: the results indicate an estimated 50 to 120 melanomas need to be removed for every life saved.

Assuming an NNT of 10 and the cost per consultation, excision and pathology to be around $300 it can be assumed, with some confidence, that the excess cost to save one life from melanoma during the period 1982–2013 as a result of enhanced secondary prevention was between $150,000 and $360,000 Australian dollars. But the NNS needs to be taken into account: the analysis above only applies to the screening of *extreme-risk* individuals where, on average, one pigmented lesion is excised at every consultation. If screening were applied to the general public, I can estimate the number of consultations per excision to be approximately 25 (if there are 25 consultations per excision, 10 excisions per melanoma diagnosis (the NNT), and 100 melanomas excised for every life saved (see Table 1), then the NNS equals 25 x 10 x 100 = 25,000. This value for the NNS has been documented elsewhere [14], and is discussed in the Introduction). If the cost of each consultation was $50, then the total cost for every excision becomes 300 + (24 x 50) = $1500, such that the cost per life saved over the period 1982–2013 balloons out to a value between $750,000 and $1.8M Australian dollars. In practice, the number of consultations per excision lay between the extreme values of 1 and 25. It is possible that costs may be recognised as prohibitive for any values greater than 5 –and particularly so if the NNT is in fact greater than 10 –suggesting that surveillance strategies can only target moderate to high-risk individuals if economic considerations are paramount. It is important to note that regular screening for melanoma will, by default, also involve screening for non-melanoma skin cancer (NMSC). However, the economic and health benefits of early detection of NMSC, at the level of the individual, are not as significant in comparison with those associated with melanoma.

Although it is impossible to assign a monetary value to any individuals’ life, I can make some interesting observations. First, the costs above should be offset against the cost of productivity–for example, if death struck at 40 years of age, then at least 25 years of employment are lost. Loss of productivity can be measured in many ways; governments would consider loss of income tax revenue an important metric. Over a period of two decades, this would amount to hundreds of thousands of dollars, given an average annual income in Australia in 2017 of $70,000 dollars. Second, the costs of prevention should be offset against the projected cost of treating more advanced melanoma, including Stages 2, 3 and 4. While the costs associated with the management of Stages 2 and 3 will be substantial, and will include, for example, those associated with sentinel node biopsy, full body scanning, surgery and radiotherapy, the majority of this cost, will, however, be determined by the costs of preventing death in the latter. Indeed, with the introduction of more expensive, and more efficacious drugs–a more efficacious drug will be more expensive than a less efficacious drug overall because it will keep the patient alive longer–the total cost per individual, with respect to the treatment of Stage 4 disease, could easily exceed $150,000 US per annum [32]. Such a sum is, on average, an order of magnitude larger than the costs associated with the management and treatment of Stages 2 and 3 [32–34]. On the other hand, the question of resource allocation arises. Could the money be better spent elsewhere? For example, it is likely more lives could be saved if the money were directed toward, say, indigenous health. Finally, the observation that melanoma is largely an affliction of the elderly means that while implementing enhanced secondary prevention may save a persons life from *melanoma*, it does nothing to prevent death from any other–and statistically more likely–cause.

The argument, however, is in favour of an enhanced secondary prevention strategy when the projections out to 2028 are taken into account. Indeed, the gains made between 1982 and 2013 are amplified: the total number of excisions begins to approach its 1982 value and the mortality rates begin to diverge significantly (Fig 6A and 6B respectively) such that the ratio of Stage 1 melanomas excised to lives saved decreases approximately sixfold (see Tables 2 and 3). Even with the assumption of an NNT of 50, the projected cost of saving a life from melanoma plummets to $400,000 at maximum (if, for example, the screening targeted a high-risk group where there were, on average, 3 consultations per excision). The question regarding the utility of enhanced secondary prevention strategies can be answered in the affirmative with confidence only if such a detection strategy is maintained beyond 2013 and the projected benefits are taken into account.

There exists an interesting symmetry, in the output of the model, between the effects of over-diagnosis and improvements in the efficacy of treating late-stage disease. While over-diagnosis increases incidence rates, it has no effect on mortality; on the other hand, improvements in the efficacy of late-stage disease improve mortality rates (at least in the short-term) while having no effect on incidence rates. The benefits of extending any given life-saving strategy beyond 2013 are apparent: while halving the likelihood of death from late-stage disease in any given year from 1982–2013 results in an average mortality reduction of 0.5 persons per year per 100,000 persons; doing the same for the period 2014–2028 results in an average mortality reduction of 0.8 persons per year per 100,000 persons. Paradoxically, by 2028 mortality rates associated with a significant improvement in the efficacy of late stage disease roughly equal the rates seen with no improvement in the efficacy of late stage disease. However, this statistic simply reflects the larger pool of patients alive with late stage disease by 2028.

I have shown that an over-diagnosis rate that increases from zero to 3 per 100 life-threatening melanomas over the period 1982 to 2013 will result in 10–20 excess excisions per 100,000 persons over the same period. For the Australian population as a whole, the result is not trivial: it equates to approximately 4000 excisions that have no impact on survival. Since the management of Stage I melanoma involves wide re-excision of the primary lesion, repeated follow-up appointments and increased anxiety, attempts should be made to address the over-diagnosis problem [35]; indeed, the biology needs to be better understood [21]. Perhaps advances in molecular diagnostics will, in the near future, be able to distinguish morphologically similar life-threatening disease from its non life-threatening counterpart.

Given that melanoma incidence rates are projected to be stable or perhaps slightly in decline, would it be a sensible option to save public money by abandoning current secondary prevention efforts? It certainly makes no sense to abandon the public health message regarding sensible sun exposure, given that this campaign is at least in part very likely to be responsible for the recent stabilization of melanoma incidence. Furthermore, it has been shown that the melanoma awareness programs implemented in Australia are cost-effective in terms of primary prevention [2]. But what does it cost to save a life with primary prevention? Shih *et al* estimate the implementation of the ‘*SunSmart*’ campaign has prevented 1000 skin cancer deaths over the years 1988–2003 in Victoria, Australia [2]. The total campaign expenditure over that period was approximately $3.44 per capita [2]. Thus with an average population of 4.5 million persons [36], and assuming 900 of those lives saved would have died from melanoma, these data suggest that to save one life from melanoma with primary prevention costs approximately $17,000 Australian dollars (which would equate to approximately $24,000 in 2017 given an average annual inflation rate since 2003 of 2.5% (tradingeconomics.com (accessed July 2017))). In contrast, I have shown that secondary prevention of death from melanoma carries a significant price tag. For the years 2013–2028, if the detection probabilities of Stage 1 melanoma are reduced to and kept at 1982 levels, and when ***p*** = 15 (the ratio of the likelihood of progression of untreated versus treated Stage 1 disease), for every life saved, the number of excess melanoma diagnoses is likely to be over 100, resulting in a cost of $800,000 Australian dollars (if the NNT is 20 and the level of secondary prevention resulted in, on average, 3 consultations per lesion excised). This large cost differential suggests secondary prevention strategies are wasteful and should be abandoned. However, there are problems associated with a sole reliance on primary prevention. First, it is clear that primary prevention strategies will have minimal impact on preventing melanoma deaths in a large proportion of the current population already at significant risk for developing melanoma (from previous sun exposure practices). Second, and as discussed previously, the promotion of primary prevention increases the NNT, which, paradoxically, is the major cause of ballooning secondary prevention costs. Finally, additional arguments have been provided that suggest the best method to reduce melanoma deaths is to focus on secondary prevention [37]. Despite these caveats, the continuation of primary prevention campaigns will, over time, as younger cohorts of sun-savvy individuals replace the population, lead to a significant reduction in the overall population risk for melanoma. The role of secondary prevention will then become less important. The costs of secondary prevention of melanoma are high. The methodology presented here should be taken as a first step in the process of providing decision-makers with useful information. Whether such information would actually lead to the withdrawal of public funding for discretionary mole removal is unknown; however, with quantitative evidence to hand, and with the ability to vary the values of input parameters, at least informed decisions–such as no change to current policy, or shifting a proportion of funding from secondary to primary prevention–can be made.

Finally, the motto ‘*prevention is better than cure*’ encapsulates conventional wisdom. But does it apply to melanoma? For the years 2013–2028 I can compare the expected number of deaths by simulating two alternative scenarios: first, by *maintaining* or *enhancing* 2013 secondary prevention levels while simultaneously *not improving* the likelihood of survival per year from the effects of late-stage disease; and second, by *abandoning* 2013 secondary prevention strategies while simultaneously *improving* the likelihood of survival in late-stage disease. What do I find? For the case ***D****(0)* = 0.05 (the initial probability of a diagnosis of Stage 1 disease per year given the presence of undiagnosed Stage 1 disease) and ***p*** = 15 (see above), there are 88 expected deaths per 100,000 persons by maintaining the secondary prevention strategy; in contrast, abandoning secondary prevention and halving the likelihood of death in any year from the effects of late-stage disease results in only 77 expected deaths per 100,000 persons. Doubling the likelihood of early detection of melanoma in 2013 and maintaining this detection rate until 2028 without changing the probability of death in any year from late-stage disease leads to 83 expected deaths per 100,000 persons. The likelihood of death in any year from late-stage disease needs only to be reduced from 0.37 to 0.30 (a reduction of 19 per cent) to equal the mortality associated with maintaining (as opposed to abandoning) the 2013 secondary prevention strategy. This analysis highlights an important and perhaps counterintuitive point: incremental improvements in the treatment of late-stage disease will, in the foreseeable future, have a comparable–and possibly greater–effect on mortality rates in comparison with those propelled by heightened surveillance and diagnostic vigilance. Yet mortality rates *per se* are not necessarily the only concern. The overall costs are similar–it was noted above that the projected cost of secondary prevention of melanoma death is of the order $400,000 dollars; on the other hand, the cost, per patient, of temporarily preventing death (over a number of years) in Stage 4 disease will also run into the hundreds of thousands of dollars. Funding considerations should include the potential effects of quality of life measures (either improvements or a deterioration) with respect to treatment of Stage 4 disease, and the observation that prevention is a ‘cure’, while Stage 4 treatment is currently palliative.
